# Graft-to-recipient weight ratio exerts nonlinear effects on prognosis by interacting with donor liver macrosteatosis

**DOI:** 10.3389/fsurg.2022.1075845

**Published:** 2023-01-17

**Authors:** Zhengtao Liu, Jingting Lyu, Xiang Li, Lu Yu, Shuping Que, Jun Xu, Lei Geng, Shusen Zheng

**Affiliations:** ^1^ Shulan International Medical College, Zhejiang Shuren University, Hangzhou, China; ^2^ NHC Key Laboratory of Combined Multi-Organ Transplantation, Key Laboratory of the Diagnosis and Treatment of Organ Transplantation, CAMS, First Affiliated Hospital, School of Medicine, Zhejiang University, Hangzhou, China; ^3^ Key Laboratory of Organ Transplantation, First Affiliated Hospital, School of Medicine, Zhejiang University, Hangzhou, China; ^4^ Shulan (Hangzhou) Hospital, Hangzhou, China; ^5^ Division of Hepatobiliary and Pancreatic Surgery, Department of Surgery, First Affiliated Hospital, School of Medicine, Zhejiang University, Hangzhou, China; ^6^ School of Medicine, Zhejiang Chinese Medical University, Hangzhou, China; ^7^ DingXiang Clinics, Hangzhou, China

**Keywords:** GWRWR, prognosis, graft failure, liver transplantation, macrosteatosis

## Abstract

**Aim:**

To investigate the interactions between the graft-to-recipient weight ratio (GWRWR) and other risk factors responsible for inferior allograft outcomes.

**Methods:**

A total of 362 patients who received liver transplantation (LT) were enrolled. Indicators such as graft/recipient weight and other prognostic factors were collected. Comparisons of indicators and survival analysis were performed in groups categorized by the GWRWR. Interactions of large-for-size grafts (LFSGs) with graft macrosteatosis (MaS) were evaluated in terms of relative excess risk caused by interaction (RERI) and attributable proportion (AP). Cytoscape visualized the role of LFSGs in the risk profile for poor prognosis.

**Results:**

Based on the GWRWR, LT cases can be categorized into three subgroups, standard (1%–2.5%), optimal (2.5%–3.0%), and inferior prognosis (>3.0%). Survival analysis confirmed clear separations in cases categorized by the above-defined limits on the GWRWR (*P* < 0.05). LFSGs caused inferior prognosis by initiating positive interactions with MaS severity.

**Conclusion:**

The GWRWR exerted nonlinear effects on prognosis in deceased donor LT cases. LFSGs (GWRWR > 3.0%) caused inferior outcomes, while grafts sized within (2.5%–3.0%) had optimal post-transplant prognosis. MaS increased the risk of poor prognosis by exerting positive synergistic effects on LFSGs.

## Introduction

Liver transplantation (LT) plays a vital role in treating end-stage liver disease. Size-mismatched LT causes severe complications that affect surgical quality and patients’ prognosis (1). Large-for-size (LFS) grafts cause inferior post-transplant prognosis via disturbed microcirculation in the liver (2). However, in small-for-size (SFS) grafts, limited microcirculatory adaptions to intensive portal flows might be responsible for poor outcomes after LT (2, 3). Of note, the graft weight-to-recipient weight ratio (GWRWR) is usually used to assess the severity of graft mismatch. Considering the weight of recipients and also the size of grafts, the GWRWR was applied as an available indicator to predict inferior prognosis caused by the small/large-for-size syndrome (SFSS/LFSS) at 0.8% and 2.5%, respectively (2). However, these cutoffs were imputed only approximately in groups by a simple binary classification of the GWRWR (4). Further studies are necessary for achieving more precise cutoffs based on continuous risk assessments between the GWRWR and the mortality rate.

As is known, LT quality is commonly determined by a network of factors comprising donors, recipients, grafts, surgical aspects, and their interactions (5). Concerns about the impacts of organ mismatch on post-transplant prognosis and its interactions with other prognostic indicators have been raised.

Therefore, in this study, a cohort including more than 360 deceased LT cases was constructed to evaluate the continuous impacts of the GWRWR on post-transplant outcomes, which might help clarify the role of graft size in risk profiles for LT patients.

## Patients and methods

### Case enrollment

The study enrolled patients who underwent liver transplantation (LT) in two liver transplant centers, first in Shulan [Hangzhou] hospital (abbreviated as SL cohort) between July 2016 and October 2017 and second in First Affiliated Hospital, School of Medicine, Zhejiang University (abbreviated as ZY cohort), between May 2020 and April 2021. The enrollment was limited to subjects who received deceased donor liver transplantation (DDLT) and ranged between adult donors and recipients (aged >18 years). Accordingly, the following types of patients were excluded: (I) adolescent donor/recipients (aged <18 years); (II) multiorgan transplantation recipients (*n* ≥ 2); (III) living donor LT.

Consent was obtained from the enrolled participants. The study was performed under the Declaration of Helsinki and approved by the ethical institutional review board of Zhejiang Shuren University.

### Data collection and disease definition

Indicators with the potential to affect post-transplant prognosis were collected for further analysis. Factors were generally categorized into the following: donor, recipient, graft, surgery, and interaction (Table 1). Routinely, recipient weights were measured before LT. Graft weights were also scaled at the end of the cold ischemia phase before implantation. Details of indicators for LT cases and prognostic information were obtained from medical record systems in each hospital.

**Table 1:** Characteristics of liver transplant cases by the GWRWR.

Characteristics	GWRWR*100			
	(0.8–2.5)	(2.5–3.0)	(3.0–4.1)	*P*-value
Number	274	53	35	
D-Age (years)	46 (33–55)	48 (42–52)	39 (29–50)	0.22
D-Gender (M,%)	216 (78.8)	42 (79.2)	31 (88.5)	0.44
D-BMI (kg/m^2^)	22.5 (20.8–24.2)	23.7 (21.6–26.0)	24.8 (22.5–27.7)	<0.05
D-Blood type (A/B/O/AB)	79/72/88/35	17/15/15/6	13/9/10/3	0.95
D-Donation type (DCD,%)	191 (69.7)	32 (60.4)	20 (57.1)	0.17
D-HBV infection (*n*,%)	28 (10.2)	5 (9.4)	0 (0)	0.12
D-HCV infection (*n*,%)	3 (1.1)	0 (0)	1 (2.9)	0.42
R-Age	52 (45–58.3)	48 (38.6–58.5)	52 (45–55.1)	0.22
R-Gender (M,%)	230 (83.9)	40 (75.5)	28 (80.0)	0.31
R-BMI (kg/m^2^)	23.5 (22.1–25.4)	22.0 (20.1–23.6)	21.1 (19.1–23.4)	<0.05
R-Blood type (A/B/O/AB)	95/67/84/28	18/14/17/4	14/9/10/2	0.97
Preoperative AFP	12.3 (3.1–13.7)	8.2 (2.4–62.7)	5.1 (2.2–30.4)	0.25
MELD score	34 (27–40)	31 (23–40)	32 (19–36)	0.10
Child–Pugh score	10 (9–11)	11 (9–11)	11 (9–12)	0.78
Primary disease				
Viral hepatitis (CHB + CHC, *n*,%)	197(71.9)	36(67.9)	21(60)	0.33
Alcoholic liver disease (*n*,%)	19 (6.9)	2 (3.8)	2 (5.7)	0.68
HCC (*n*,%)	148(54.0)	26(49.1)	13(37.1)	0.16
Liver failure (*n*,%)	67(24.5)	14(26.4)	8(22.9)	0.93
Primary biliary cirrhosis (*n*,%)	6 (2.2)	3 (5.7)	1 (2.9)	0.08
G-Weight (g)	1,330 (1,146–1,484)	1,626 (1,440–1,809)	1,790 (1,625–2,090)	<0.05
G-MaS (yes,%)	77(28.1)	23(43.4)	21(60.0)	<0.05
CIT (min)	589 (451–740)	580 (454–785)	570 (405–712)	0.68
WIT (min)	10 (4–17)	8 (2–15)	10 (2–16)	0.37
Surgical duration (min)	297 (265–347)	285 (250.5–330.5)	338.5 (288–373)	0.01
Blood transfusion				
pRBC (U)	4 (0–7.5)	4 (2–8)	6 (2.5–8)	0.04
FFP (ml)	810 (656–1,013)	803 (618–1,055)	905 (660–1,240)	0.04
Blood loss (ml)	1,100 (800–2,000)	1,200 (800–1,800)	1,500 (1,150–2,125)	0.03
SLT (*n*,%)	8(2.9)	3(5.7)	1(2.9)	0.59
EAD (*n*,%)	62 (22.6)	16 (30.2)	11 (31.4)	0.31
PNF (*n*,%)	8 (2.9)	1 (1.9)	3 (8.6)	0.18
Post-transplant variables				
ICU stay (days)	14(10.6–17.8)	13.0(8.6–17.8)	12.8(6.5–15.5)	0.15
Peak ALT level (U/L)	1,300(501–1,700)	1,781(871–2,417)	2,254(693–2,724)	<0.01
Peak AST level (U/L)	3,260 (935–4,419)	4,907 (1,667–5,963)	6,842 (1,307–8,830)	<0.01
Peak TB level (μmol/L)	216(68–290)	237(77–359)	196(85–293)	0.65
Time from LT to the end of follow-up (days)	693 (499–781)	671 (472–774)	685 (490–761)	0.29
ABO mismatch (*n*,%)	28 (10.2)	4 (7.5)	3 (8.6)	0.81
GWRWR*100	2.0 (1.7–2.2)	2.7 (2.6–2.8)	3.2 (3.1–3.3)	<0.05

Quantitative data are presented as median (IQR) and compared by using the Mann–Whitney *U* test; Categorical variables are presented by number and percentage in the whole cohort and compared by using the *χ*^2^ test.

ALT, alanine aminotransferase; AFP, alpha-fetoprotein; AST, aspartate aminotransferase; BMI, body mass index; CHB, chronic hepatitis B; CHC, chronic hepatitis C; D, donor; DBD, donation after brain death; DCD, donation after circulatory death; EAD, early allograft dysfunction; F, female; FFP, fresh frozen plasma; G, graft; GF, graft failure; GW, graft weight; GWRWR, graft weight-to-recipient weight ratio; HCC, hepatocellular carcinoma; HR, hazard ratio; ICU, intensive care unit; IQR, interquartile range; M, male; MaS, macrosteatosis; MELD, model for end-stage liver disease; PD, patient death; PNF, primary non-function; pRBC, packed red blood cells; R, recipient; RW, recipient weight; SLT, split liver transplantation; TB, total bilirubin.

Macrosteatosis (MaS) was assessed under microscopic observation in a double-blinded manner (6). Graft failure (GF) and patient death (PD) were the primary endpoints representing the prognosis. The model for end-stage liver disease (MELD) score was calculated by using the formula provided in a previous study (7). Child–Pugh scores were calculated based on the clinical factors of total bilirubin, albumin, prothrombin time, ascites, and encephalopathy (8). Diagnosis of early allograft dysfunction (EAD) was defined according to criteria updated in a prior study (9). Primary non-function (PNF) was defined as impaired liver function needing re-transplantation in an emergency ward within 72 h after LT (10).

### Statistic analysis

Categorized by the GWRWR, quantitative data were presented as median [interquartile range (IQR)] and compared by using the Mann–Whitney *U* test. A comparison of the distribution of qualitative data in different groups was performed by using the *χ*^2^ test. The hazard ratios (HRs) of sectionalized GWRWR and other potential indicators of GF/PD occurrence were assessed by using univariate and multicovariate COX regression models. Specifically, the HRs for prognosis were assessed between the selected and the remaining groups. Furthermore, graft/patient survival (GS/PS) rates were compared by using the log-rank test across groups by GWRWR classifications. Relative excess risk caused by interaction (RERI) and attributable proportion (AP) was applied to investigate the synergistic effects on LT prognosis for graft size with other relevant factors. To be specific, RERI, AP, and their confidence intervals (CIs) were calculated based on regression coefficients (B) and correlated covariance from different comparisons via scale provided in previous literature (11). RERI > 0 and AP > 0 meant positive interaction (11). The network was constructed by using Cytoscape (v3.9.0) to visualize the associations between the GWRWR and LT prognosis within risk profiles speculated from survival analysis.

## Results

### Clinical features of LT cases

**Figure 1:**
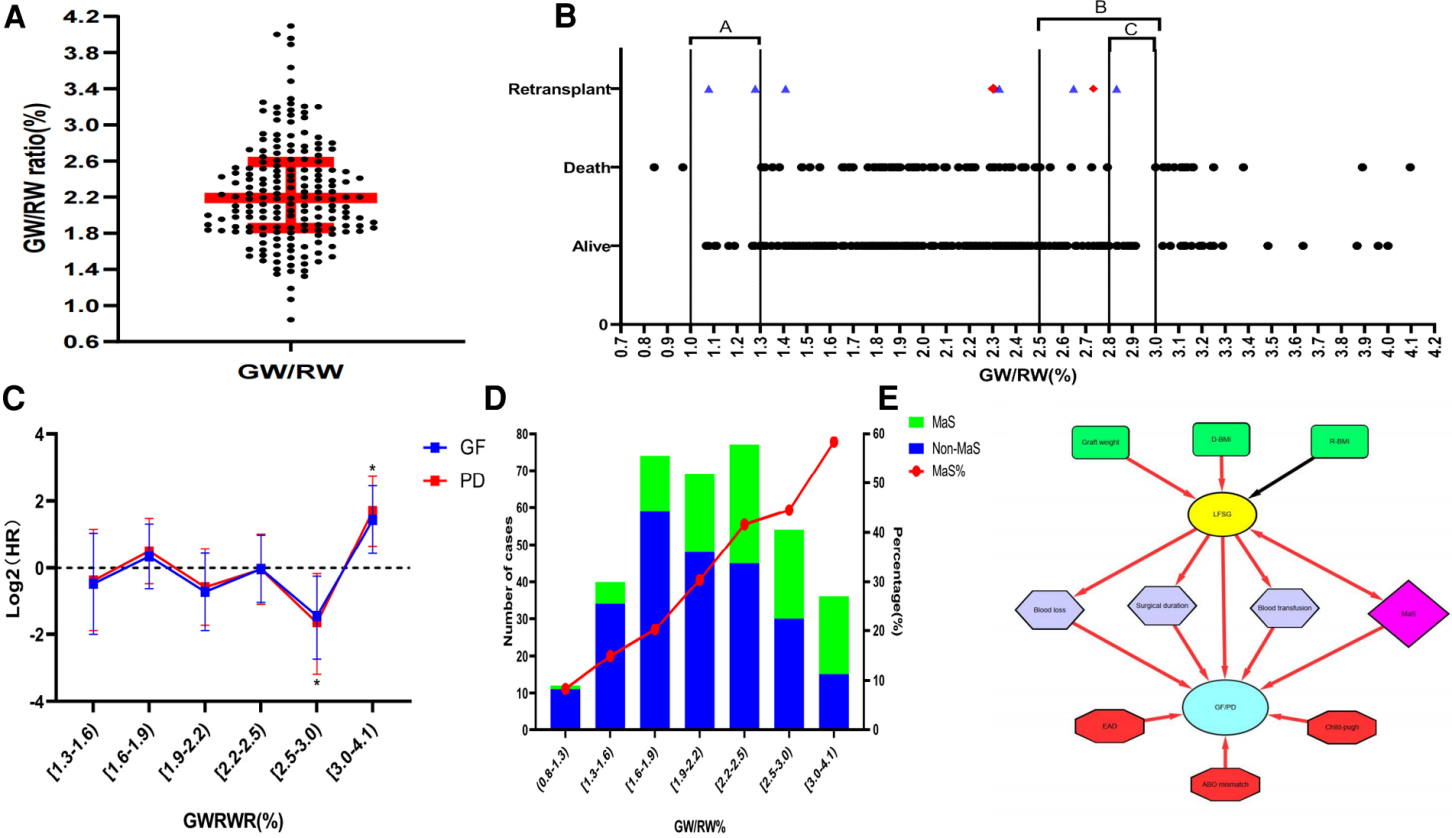
Impact of the GWRWR on prognosis by interaction with graft MaS. (**A**) Distribution of the GWRWR for LT cases in the whole cohort; (**B**) scatter diagram on the association between the GWRWR and prognosis; the points marked in red denote final deaths after re-LT during follow-up, and the points marked in blue denote survival during follow-up; (**C**) continuous-adjusted HRs of the GWRWR on the prognosis of patients after LT; HRs were assessed by using the COX regression model by adjusting donor/recipient BMI and graft MaS; *represents significant differences compared with the other groups; (**D**) correlation between the GWRWR and MaS prevalence; the left *Y*-axis represents the number of MaS/non-MaS cases, and the right *Y*-axis represents the percentage of MaS in the corresponding subgroup; (**E**) schematic diagram showing the complex role of LFSGs in the risk profile for poor prognosis after LT; the red line denotes positive correlations on both sides, and the black line denotes negative correlations on both sides. BMI, body mass index; GWRWR, graft weight-to-recipient weight ratio; HR, hazard ratio; LFSG, large-for-size graft; LT, liver transplantation; MaS, macrosteatosis.

A total of 362 LT cases included for 354 recipients were enrolled for analysis. Our study enrolled 243 cases from the SL cohort and 119 cases from the ZY cohort, accounting for 85.9% and 70.8% of all adult LT cases during the same period in each center. Eight patients underwent re-transplantation, and six remained alive at the end of the follow-up. The median of the GWRWR for the whole cohort was 2.15% with IQR (1.79%–2.49%) (Figure 1A). Most re-transplant patients used smaller (GWRWR < 1.5) or larger (GWRWR > 2.5) grafts during the first LT (Figure 1B).

LT cases were divided into three subgroups by the GWRWR at 2.5% and 3.0% (Table 1). As expected, body mass was higher in donors but lower in recipients, followed by GWRWR increment stepwise. The GWRWR was positively affected by graft weight. A sharp MaS increment was observed with GWRWR elevation (8.3% in the group with a lower GWRWR and 58.3% in the group with a higher GWRWR). Meanwhile, increased post-transplant liver enzymes [including alanine/aspartate aminotransferase (ALT/AST)], higher blood loss, and a higher volume of transfusions indicated more severe transplant complications in those who received LFS grafts (GWRWR > 3.0). Because of insignificant intergroup differences in recipient disease severity (similar MELD scores and primary disease), it was more challenging to treat LT patients using large-for-size grafts (LFSG, GWRWR ≥ 3.0%), and challenges were in the form of higher blood loss, higher transfusion volume, and longer surgical duration (*P* < 0.05, Table 1). Besides, it was comparable for other clinical indicators in groups categorized by GWRWR. More details about clinical indicators are given in Table 1.

### Nonlinear impact of the GWRWR on post-transplant prognosis

Distribution of survival status for all enrolled subjects in the follow-up duration is presented in Figure 1B. No death occurred in patients whose GWRWR ranged between (2.8%–3.0%) and (1.0%–1.3%). COX regressions revealed that the LFSGs caused an inferior prognosis compared with the remaining groups.

**Figure 2:**
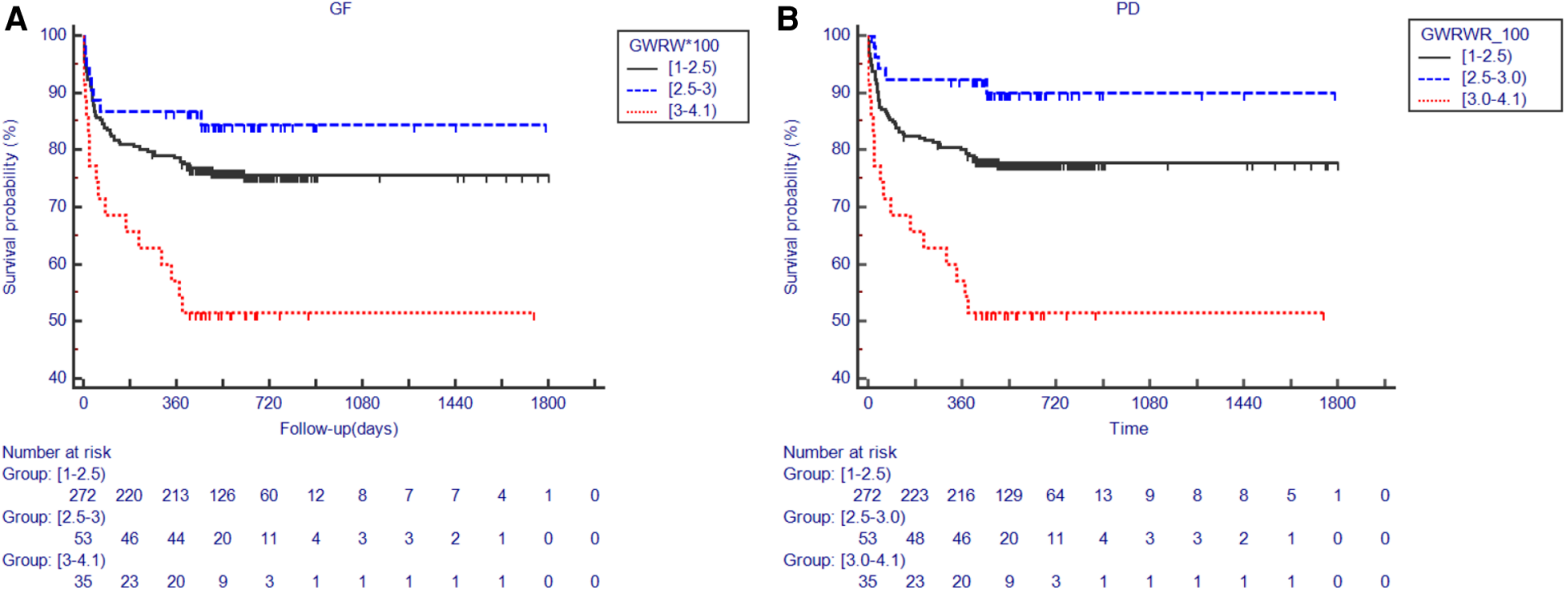
Kaplan–Meier plot for post-transplant outcomes in groups categorized by the GWRWR. (**A**) Comparisons on the GS rate categorized by the GWRWR; numbers at risk for each group with different GWRWRs in different periods presented under the Kaplan–Meier plot; (**B**) comparisons on the PS rate categorized by the GWRWR; numbers at risk for each group with different GWRWRs in different periods presented under the Kaplan–Meier plot. *represents significant differences compared with the group using grafts with a GWRWR between 1.0% and 2.5%. GS, graft survival; GWRWR, graft weight-to-recipient weight ratio; PS, patient survival.

Most patients (75%) used grafts with the GWRWR ranging between 1.0% and 2.5%. An insignificant HR was observed in the group with a lower intraheterogeneity (Figure 1C). A GWRWR between 2.5% and 3.0% seemed to be the most suitable with a lower risk of GF/PD. Moreover, these results remained consistent after adjusting for correlated donor/recipient BMI and graft MaS (Figure 1C and Table 2). Furthermore, survival curves also confirmed clear separations in groups with different GWRWRs (Figure 2). Considering the separated survival curves in groups categorized by the GWRWR, we defined the three groups [(1%–2.5%), (2.5%–3.0%), (3.0%–4.1%) on GWRWR] to have “normal,” “optimal,” and “inferior post-transplant prognoses, respectively. LT by SFSS organ (defined by GWRWR < 1%) seemed unsuitable, as highlighted by the absence of survivors during the follow-up period. However, the result was uncertain for a few cases (only two) reported in this cohort (Figure 1B).

**Table 2:** Survival and interactive analyses between the GWRWR and prognostic indicators.

Characteristics	Survival analysis		Interactive analysis	
	HR for GF (95% CI)	HR for PD (95% CI)	RERI (95% CI)	AP (95% CI)
D-Age (per year)	0.99 (0.98–1.01)	0.99 (0.98–1.01)		
D-Gender (M vs. F)	1.32 (0.74–2.34)	1.19 (0.65–2.20)		
D-BMI (per kg/m^2^)	1.00 (0.92–1.09)	0.99 (0.91–1.09)		
D-Donation type (DCD vs. DBD)	1.38 (0.78–2.45)	1.39 (0.77–2.50)		
D-HBV infection (Y vs. N)	0.83 (0.40–1.73)	0.77 (0.35–1.68)		
D-HCV infection (Y vs. N)	0.81 (0.11–5.83)	0.87 (0.12–6.28)		
R-Age (per year)	0.99(0.98–1.01)	1.00(0.98–1.02)		
R-Gender (M vs. F)	1.01 (0.59–1.73)	1.04 (0.60–1.82)		
R-BMI (per kg/m^2^)	0.98 (0.92–1.05)	0.99 (0.93–1.06)		
Preoperative AFP (per 100 ng/ml)	1.00 (0.99–1.01)	1.00 (0.99–1.01)		
MELD score	1.01 (0.99–1.04)	1.01 (0.99–1.04)		
Child–Pugh score	1.24 (1.10–1.40)*	1.25 (1.10–1.41)*		
Primary disease				
Viral hepatitis (CHB + CHC,Y vs. N)	0.80(0.52–1.24)	0.94(0.59–1.49)		
Alcoholic liver disease (Y vs. N)	0.30 (0.07–1.22)	0.33 (0.08–1.36)		
HCC (Y vs. N)	0.90 (0.60–1.35)	0.86 (0.56–1.32)		
Liver failure (Y vs. N)	1.35(0.86–2.11)	1.36(0.85–2.18)		
Primary biliary cirrhosis (Y vs. N)	1.07 (0.34–3.37)	0.72 (0.18–2.92)		
G-Weight (per 100 g)	1.02 (0.95–1.08)	1.02 (0.96–1.09)		
G-Steatosis				
Type (MaS vs. None-MaS)	1.33 (1.09–1.64)*	1.31 (1.06–1.63)*		
Degree for MaS (per 10%)	1.27 (1.05–1.52)*	1.22 (1.01–1.48)*		
CIT (per hour)	1.00 (0.99–1.01)	1.00 (0.99–1.01)		
WIT (per minute)	0.99 (0.98–1.01)	0.99 (0.98–1.01)		
Surgical duration (per hour)	1.18 (1.02–1.36)*	1.19 (1.02–1.38)*		
Blood transfusion				
pRBC (per U)	1.05 (1.03–1.07)*	1.05 (1.03–1.07)*		
FFP (per 100 ml)	1.05 (1.01–1.10)*	1.06 (1.03–1.10)*		
Blood loss (per 100 ml)	1.03 (1.02–1.04)*	1.03 (1.02–1.04)*		
SLT (Y vs. N)	1.48(0.55–4.05)	1.65(0.60–4.50)		
EAD (Y vs. N)	3.12 (2.06–4.71)*	2.80 (1.81–4.33)*		
PNF (Y vs. N)	NA	11.68 (5.81–23.5)*		
Post-transplant variables				
ICU stay (per 3 days)	1.06(1.01–1.10)*	1.03(0.99–1.08)		
ABO mismatch (Y vs. N)	5.10 (3.21–8.10)*	5.15 (3.18–8.34)*		
GW/RW ratio*100	1.13 (0.78–1.65)	1.19 (0.81–1.75)		
GW/RW*100				
(1.0–1.3)	0.73 (0.18–2.94)	NA		
(1.3–2.5)	0.92 (0.74–1.15)	1.01 (0.80–1.28)		
(2.5–3)	0.85 (0.67–1.06)	0.75 (0.57–0.96)*		
(3.0–4.1)	1.20 (1.05–1.38)*	1.24 (1.08–1.43)*		
LFSG + MaS (Y vs. N)	2.65 (1.37–5.11)*	3.01 (1.55–5.81)*		
LFSG + MaS on GF			2.92 (0.66–5.20)	0.73 (0.41–1.06)
LFSG + MaS on PD			3.33 (0.78–5.88)	0.72 (0.41–1.02)
Optimal grafts + MaS on GF			−1.02 (−3.12/1.08)	−1.59 (−5.15/1.98)
Optimal grafts + MaS on PD			−1.68 (−4.11/0.75)	−3.77 (−9.94/2.39)

HRs were evaluated by using the COX regression model; for dichotomous covariates, HRs were evaluated by categorical comparison; for continuous covariates, HRs were evaluated per 1 unit increment of exposure (in bracket); biological interactions were evaluated by using RERI and AP.

AFP, alpha-fetoprotein; AP, attributable proportion; BMI, body mass index; CHB, chronic hepatitis B; CHC, chronic hepatitis C; D, donor; DBD, donation after brain death; DCD, donation after circulatory death; EAD, early allograft dysfunction; F, female; FFP, fresh frozen plasma; G, graft; GF, graft failure; GW, graft weight; GWRWR, graft weight-to-recipient weight ratio; HCC, hepatocellular carcinoma; HR, hazard ratio; ICU, intensive care unit; IQR, interquartile range; M, male; MaS, macrosteatosis; MELD, model for end-stage liver disease; PD, patient death; PNF, primary non-function; pRBC, packed red blood cells; R, recipient; RERI, relative excess risk due to interaction; RW, recipient weight; SLT, split liver transplantation.

* Represented a significant difference in HRs at *P* < 0.05.

### Interactions of the GWRWR with other prognostic indicators

Despite its close connection with the GWRWR, MaS also increased the risk of PD/GF in patients after LT. A further interactive analysis found that MaS exerted additive effects on LFSGs to cause poor prognosis with positive RERI and AP. But in the group with optimal prognosis, the GWRWR caused a lower GF/PD risk by initiating a negative interaction with MaS (Table 2). Meanwhile, surgical indicators [blood loss, pRBC/fresh frozen plasma (FFP) transfusion, and operational duration] associated with LFSGs also showed positive associations with inferior prognosis (Table 2). In addition, factors such as a higher Child–Pugh score, EAD status, and ABO mismatch were associated with poor prognosis (Table 2). However, these factors were less correlated with GWRWR variation (Table 1).

### Diagram of the interactive risk profile for LT

The profile of the risk factors for LT recipients is summarized in Figure 1E. The GWRWR was commonly determined by factors such as recipient/donor BMI, graft weight, and MaS status. LFSGs increased GF/PD risk by causing more surgical complications (prolonged surgical duration, higher blood loss/transfusion volume) and synergistic effects with concomitant graft MaS. The GWRWR was less related to the potential prognostic factors of the recipients (Child–Pugh score, EAD status) and to donor/recipient interaction (ABO mismatch).

## Discussion

Safety cutoffs for the GWRWR on LT prognosis are defined at 2.5%, with controversies surrounding the rate across previous studies (3). However, in our study, an inconsistent distribution of deaths was observed in LT patients who received grafts. In a cohort of 362 LT patients, we found that graft size exerted a nonlinear effect on prognosis. Grafts with a GWRWR between 2.5% and 3.0% had optimal prognoses. Defined by a GWRWR ≥ 3.0%, LFSGs caused inferior post-transplant prognosis by initiating positive interactions with graft MaS, leading to concerns about the coexistence of LFSGs with MaS.

MaS is a well-known risk factor in the LT process (6, 12). However, the connection and interactive effects between MaS and graft size have been rarely reported in previous studies. In this study, a higher GWRWR denoted a higher occurrence of MaS, especially for LFSGs. MaS impaired graft function by initiating a positive interaction with LFSGs. Recent guidelines have also emphasized the importance of pathological examinations on larger organs before the performance of LT (13). Accordingly, more caution should be exercised with regard to the coexistence of LFSGs with MaS.

LT by LFSG poses a challenge to surgeons in the form of potential additional operations to avoid LFSS (graft size reduction/abdomen opening) (3). In our study, a larger-sized liver caused an inferior post-transplant prognosis in the form of more severe surgical complications (higher blood loss and prolonged surgical durations) (2). Moreover, the complex surgical process might aggravate graft damage from prolonged cold ischemia (14). Combined with the advances in surgical technology, strict controls on surgical duration might be an effective approach to avoid the risk of LFSGs on prognosis. However, this hypothesis should be validated in a study with a prospective design. To sum up, LFSGs affected LT prognosis by interacting with factors related to graft and surgery. In contrast, the association with prognostic factors related to recipients (Child–Pugh) and D/R interaction was less (ABO mismatch) (Figure 1E).

The safety threshold for the GWRWR saw an increase (<2.5%) in DDLT patients (4). However, the results were ambiguous for making even an approximate dichotomous comparison in a few subjects. Fukazawa speculated that the rational curve for determining the risk of graft size in prognosis should be “U”-shaped with an optimal range in the middle (2). In our study, an inconsistent distribution of deaths was observed in LT patients who received grafts with a GWRWR > 2.5%. We found that the risk cutoff for the GWRWR could be defined at >3.0%. In contrast, the optimal interval (between 2.5% and 3.0%) occurred close to the risk peak with HR valley. Similar trends presented in the lower end (no survivors in the group with a GWRWR < 1%, but no deaths in the group with a GWRWR between 1% and 1.3%) with statistical insignificance for a few cases. Insufficient tissue perfusion was a common feature for mismatched organs (2). We speculated that the “slightly bigger” grafts (a GWRWR ranging between 2.5% and 3.0%) might be stimulated to improve organ quality (e.g., regeneration) under tolerated surviving stress. As an available tool, plans for a multiomic study on LT patients are on the anvil to reveal the rationale underlying the complex association between the GWRWR and prognosis.

Despite the novel findings in this study, limitations exist and should be placed as follows: First, factors such as donation after circulatory death (DCD) might exert confounding effects on the association between the GWRWR and prognosis. Second, there is the possibility of bias with regard to the association between graft size and prognosis in the form of different operation times and LT centers. Third, the cause of GF was not ascertained specifically for each recipient. Another etiology study might help clarify the impact of size-mismatched LT on poor prognosis. Fourth, fewer LT patients received LFSGs (<10%) and they were enrolled in the whole cohort. Moreover, our results should be further validated in practice with more subjects receiving mismatched graft sizes. Fifth, inconsistent criteria on graft-recipient match might also cause a systemic disparity in results from different LT centers. To sum up, the abovementioned defects might exert confounding effects on associations between mismatched grafts and post-transplant prognosis. Further prospective study designs involving more LT patients with LFSGs might help confirm the extent of risk in post-transplant prognosis.

In conclusion, the GWRWR exerted nonlinear effects on post-transplant prognosis in DDLT patients. LFSGs (GWRWR > 3.0%) exerted a positive risk for poor prognosis, while grafts ranging between 2.5% and 3.0% had optimal prognoses. Graft MaS increased the risk for poor prognosis by exerting positive synergistic effects on LFSGs. Further mechanistic studies might help explain the prognostic gaps between groups with adjacent tissue graft sizes.

## Data Availability

The original contributions presented in the study are included in the article/Supplementary Material, and further inquiries can be directed to the corresponding author/s.
